# The Parasite in Science

**Published:** 1896-03

**Authors:** 


### Editorial.

### THE PARASITE IN SCIENCE.

Among the lower forms of life, bacteriology informs us, are to be found the saprophytic bacteria, living solely on dead matter, bringing into active work new elements, which in turn develop new forces, and make possible the constant increase of animal and vegetable forms. "Deprive higher vegetation of the carbon and nitrogen supplied to it as a result of bacterial activity, and its development comes rapidly to an end; rob the animal kingdom of the food-stuffs supplied to it by the vegetable world, and life is no longer possible." These, then, are the beneficent forms of lowly life. They constitute the largest number of bacteria upon the surface of the globe. There exists another class that live a parasitic life, feeding on living organisms and constituting the forms which go to make up most of the diseases described in our pathological works.

This illustration from the forms lowest in the scale of life is characteristic of all stages of existence, from the flora in the forest to the fauna in the African jungle. Man, with his usual lordly assumption of superiority, ordinarily regards these with contemptuous indifference, but realizes growth or death, through their influence, equally with more humble creations.

That which is true in the physical world seems to be equally true in the mental, and the more we attempt to examine the psychical phenomena are we impressed with the remarkable similarity and universality of the laws governing each.

Life and death are the active presentations in all physical existence; the beginning and the end of organized being. The child grows to the man, and the man to old age, and then comes disintegration and death. First the active life-force combating disease; then the period of weakness, the increased activity of parasites, followed by death; and then the beneficent work of the saprophytic bacteria, bringing the dead matter into new forms. Thus may be classed the orderly arrangement of physical life, and the most critical cannot find any fault with it. It is all necessary for the prolongation of the activities of the world, and without it the wheels of progress, in a physical sense, would cease to move.

While man has the power of reasoning and voluntary movements there would seem to be a limit to the action of this law, and that it need not be extended to the mental processes. The products of the human intellect are not supposed to die, nor to require a mental saprophyte to bring them into new forms. The mental labor of the world lives in its creations, and upon these foundations other mental superstructures will continue to be erected.

It is difficult to understand why man should imitate the lower vegetable and animal forms, unless this comes through the hereditary taint of evolution, but we do know that in one particular at least the parasitic tendency lives with the race, and that with a pertinacity that would do credit to a more worthy object.

We have had the plagiarist with us from time immemorial, and we have repeatedly called attention to this blot upon our profession, but the subject is by no means exhausted, for the plagiarist is still a living factor among us, stealing the work of the brains of men and doing all the harm possible in his worthless career. It is not with him, however, that we wish at present to deal, but with one very near in kinship,—the parasite of original thought. It may seem strange that any distinction should be made between the two, the plagiarist and the parasite, but when the result of the work of these two is analyzed it will be found that, while the effects are practically the same with both, the method of work is quite different. The plagiarist fastens upon the completed work and takes it for his own, and with a few alterations, or none at all, as may suit him best, starts it again upon the world under his own name. The scientific parasite is one who is ever on the watch for original ideas from other men. He does not possess the power to generate these in his own mental organization, and hence must seek pabulum elsewhere. With a keenness of vision which does him infinite credit, he will discover an original thought not observable by other men. This, once caught, is taken and elaborated, and in its new dress is spread before astonished audiences as a fine piece of original work. If the originator of this idea be mentioned at all, it is in such an indifferent way that the glorious halo around the new aspirant for favor is not in the least dimmed. We are constantly reminded in our professional reading of the efforts of some to appear great on the capital furnished by others. The evil would not be so serious if these men would be as frank as Edison when speaking of the Roentgen process; that while all honor was due the discoverer, he proposed to try and make it a commercial success. In the case of Roentgen no amount of parasitic work can destroy or even affect the great results attained by the discoverer, and hence the changes rung upon it, and the efforts to advance, through the activity of many minds, will only result in good.

This, however, is not true with a large portion of the world's work. The parasite is ever with us, and almost all original investigators suffer by his detestable presence. Dentistry is full of this class. While this profession has probably more original workers than any other, it has in its ranks an equal proportion of men more or less intellectually gifted who have made their entire reputations upon the faculty possessed by them of taking other men's work, repeating the experiments, and then boldly giving it all out as practically new. We think it is time that the dental profession should place its mark of condemnation upon these, and let the plagiarist and the parasite in science go down into oblivion together.

